# Cardiac Piezo1 Exacerbates Lethal Ventricular Arrhythmogenesis by Linking Mechanical Stress with Ca^2+^ Handling After Myocardial Infarction

**DOI:** 10.34133/research.0165

**Published:** 2023-06-09

**Authors:** Sheng-an Su, Yuhao Zhang, Wudi Li, Yutao Xi, Yunrui Lu, Jian Shen, Yuankun Ma, Yaping Wang, Yimin Shen, Lan Xie, Hong Ma, Yao Xie, Meixiang Xiang

**Affiliations:** ^1^ Department of Cardiology, Cardiovascular Key Laboratory of Zhejiang Province, The Second Affiliated Hospital, Zhejiang University School of Medicine, Hangzhou 310009, China.; ^2^ Department of Endocrinology, The Second Affiliated Hospital, Zhejiang University School of Medicine, Hangzhou 310009, China.; ^3^ Texas Heart Institute, Houston, TX 77030, USA.

## Abstract

Ventricular arrhythmogenesis is a key cause of sudden cardiac death following myocardial infarction (MI). Accumulating data show that ischemia, sympathetic activation, and inflammation contribute to arrhythmogenesis. However, the role and mechanisms of abnormal mechanical stress in ventricular arrhythmia following MI remain undefined. We aimed to examine the impact of increased mechanical stress and identify the role of the key sensor Piezo1 in ventricular arrhythmogenesis in MI. Concomitant with increased ventricular pressure, Piezo1, as a newly recognized mechano-sensitive cation channel, was the most up-regulated mechanosensor in the myocardium of patients with advanced heart failure. Piezo1 was mainly located at the intercalated discs and T-tubules of cardiomyocytes, which are responsible for intracellular calcium homeostasis and intercellular communication. Cardiomyocyte-conditional Piezo1 knockout mice (Piezo1*^Cko^*) exhibited preserved cardiac function after MI. Piezo1*^Cko^* mice also displayed a dramatically decreased mortality in response to the programmed electrical stimulation after MI with a markedly reduced incidence of ventricular tachycardia. In contrast, activation of Piezo1 in mouse myocardium increased the electrical instability as indicated by prolonged QT interval and sagging ST segment. Mechanistically, Piezo1 impaired intracellular calcium cycling dynamics by mediating the intracellular Ca^2+^ overload and increasing the activation of Ca^2+^-modulated signaling, CaMKII, and calpain, which led to the enhancement of phosphorylation of RyR2 and further increment of Ca^2+^ leaking, finally provoking cardiac arrhythmias. Furthermore, in human induced pluripotent stem cell-derived cardiomyocytes (hiPSC-CMs), Piezo1 activation remarkably triggered cellular arrhythmogenic remodeling by significantly shortening the duration of the action potential, inducing early afterdepolarization, and enhancing triggered activity.This study uncovered a proarrhythmic role of Piezo1 during cardiac remodeling, which is achieved by regulating Ca^2+^ handling, implying a promising therapeutic target in sudden cardiac death and heart failure.

## Introduction

Despite the improved prognosis of cardiovascular diseases, the prevalence of sudden cardiac death (SCD) keeps rising. Coronary artery disease often leads to cardiac structural and electrical remodeling due to myocardial ischemia or infarction. Such extensive cardiac remodeling eventually precipitates pump failure and fatal ventricular arrhythmias. In the setting of myocardial infarction (MI), the structural and electrical remodeling initially helps in compensating cardiac performance. But over time, the persistent ischemia gradually elevated sympathetic tone, and ongoing inflammation caused progressive ventricular enlargement and fibrosis, leading to adverse alteration of cardiac mechanical properties including elevated end-diastolic pressure and so on [1]. Although it has been noted that increased ventricular pressure results in the appearance of early afterdepolarization (EAD) and potential electrical instability for decades [2], the direct linkage between mechanical stress and arrhythmia formation is still lacking. Meanwhile, ion channel-mediated electrical remodeling, including action potential and intracellular calcium cycling, contributes to ventricular arrhythmogenesis [3]. Thus, there has been a growing interest in mechano-modulated ion channel remodeling as a therapeutic target for MI with implications of heart failure and SCD.

The recently discovered protein Piezo1 is a mechanosensitive cation channel. It appears like a triskelion with an ionic pore in the middle, displaying nonselective cation permeability with a greater preference to Ca^2+^. It serves as a sensor to transduce mechanical forces into physiological effects via transmembrane Ca^2+^ flux [4,5]. The cardiovascular relevance of Piezo1 initially rises to prominence in endothelial responses to mechanical stress, with studies illustrating that endothelium–Piezo1 plays critical roles in vascular maturation [6,7] and blood pressure modulation [8,9]. Besides, the importance of smooth muscle Piezo1 in vascular remodeling is elegantly demonstrated. Piezo1 opening in smooth muscle cells leads to a rise in cytosolic Ca^2+^ and further activation of transglutaminases, showing a trophic effect on resistance arteries in hypertension [10]. Moreover, the expression of Piezo1 is not restricted to endothelial cells and smooth muscle cells. It has also been implicated in the mechanosensing of cardiac fibroblasts [11], cardiomyocytes [12,13], and inflammatory cells [14,15]. Recently, we and Xiao group have demonstrated that cardiac Piezo1 functions as a key component of mechanosensing machinery in regulating heart maturation and pathological hypertrophy [12,13]. Activated Piezo1 channel by mechanical stretching generates extracellular Ca^2+^ influx and provokes mechano-chemo-transduction including calcineurin/calpain and reactive oxygen species (ROS) pathway, which eventually leads to cardiomyopathy and heart failure. It may provide a novel promising therapeutic target for heart diseases.

Given the association between mechanical pressure and cardiac electrical instability, we hypothesize that Piezo1 may mediate mechanical stretch-induced arrhythmogenesis and adverse cardiovascular events following MI. Here, we investigate the relationship between Piezo1 expression and human heart dysfunction. By applying mouse genetics and pharmacological intervention, we aim to demonstrate the function of Piezo1 in cardiomyocytes after MI and explore its role in cardiac remodeling after myocardial injury.

## Results

### Piezo1 is up-regulated in human failing hearts

**Fig. 1.**
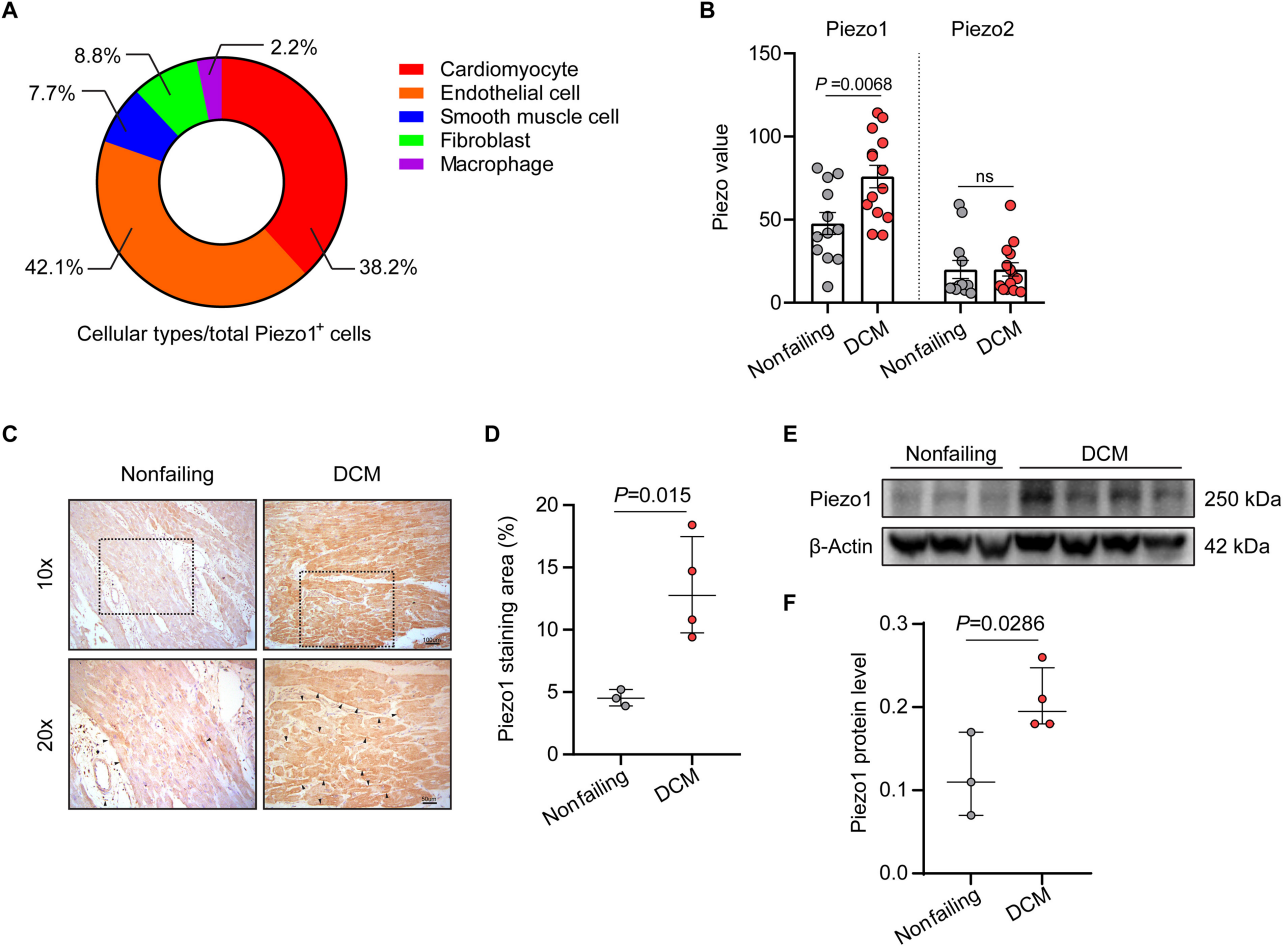
Piezo1 is up-regulated in human failing hearts. (A) Pie chart illustrating the ratio of cellular types in total Piezo1^+^ cells in heart by re-analyzing the human single-cell sequencing data from GSE109816. (B) Quantified value of Piezo1 and Piezo2 RNA expression in nonfailing human hearts and hearts from patients with DCM according to the RNA-sequencing data from GSE29819. (C) Representative images of immunohistochemical staining of Piezo1 in the anterior wall of the left ventricle from a nonfailing heart and a heart from a patient with DCM; black arrows indicate Piezo1 immunopositive areas; scale bars represent 100 and 50 μm, respectively. (D) Piezo1 staining area/total area was calculated. (E) Piezo1 expression in the left anterior wall from nonfailing hearts (*n* = 3) and hearts from patients with DCM (*n* = 4) was detected via Western blotting, with β-actin used as a loading control. (F) Piezo1 protein expression was quantified according to the immunoblot.

Although Piezo1 is widely considered to be expressed in the cardiovascular system, its distribution among different cell types in the heart remains elusive. By re-analyzing the single-cell sequence data of the normal human adult heart [Gene Expression Omnibus (GEO) accession no: GSE109816] [16], we discovered that cardiomyocytes accounted for 38.2% of Piezo1^+^ cells in the human heart, one of the most abundant sources. The remaining Piezo1^+^ cells were distributed in endothelial cells (42.1%), fibroblasts (8.8%), smooth muscle cells (7.7%), and macrophages (2.2%) (Fig. 1A). In the human diseased hearts, the RNA-sequencing data from human failing hearts with dilated cardiomyopathy (DCM) (GEO accession no: GSE29819) showed that the mRNA level of Piezo1 was up-regulated in the failing heart, while Piezo2 expression and other key ion channels remained unchanged (Fig. 1B and Fig. S1A). To further validate these findings, we used immunohistochemical staining (Fig. 1C and D) and Western blotting (Fig. 1E and F) to confirm that Piezo1 was significantly increased in the myocardium of patients with advanced heart failure of DCM.

### Cardiomyocyte-specific deficiency of Piezo1 improves cardiac function after MI in mice

**Fig. 2.**
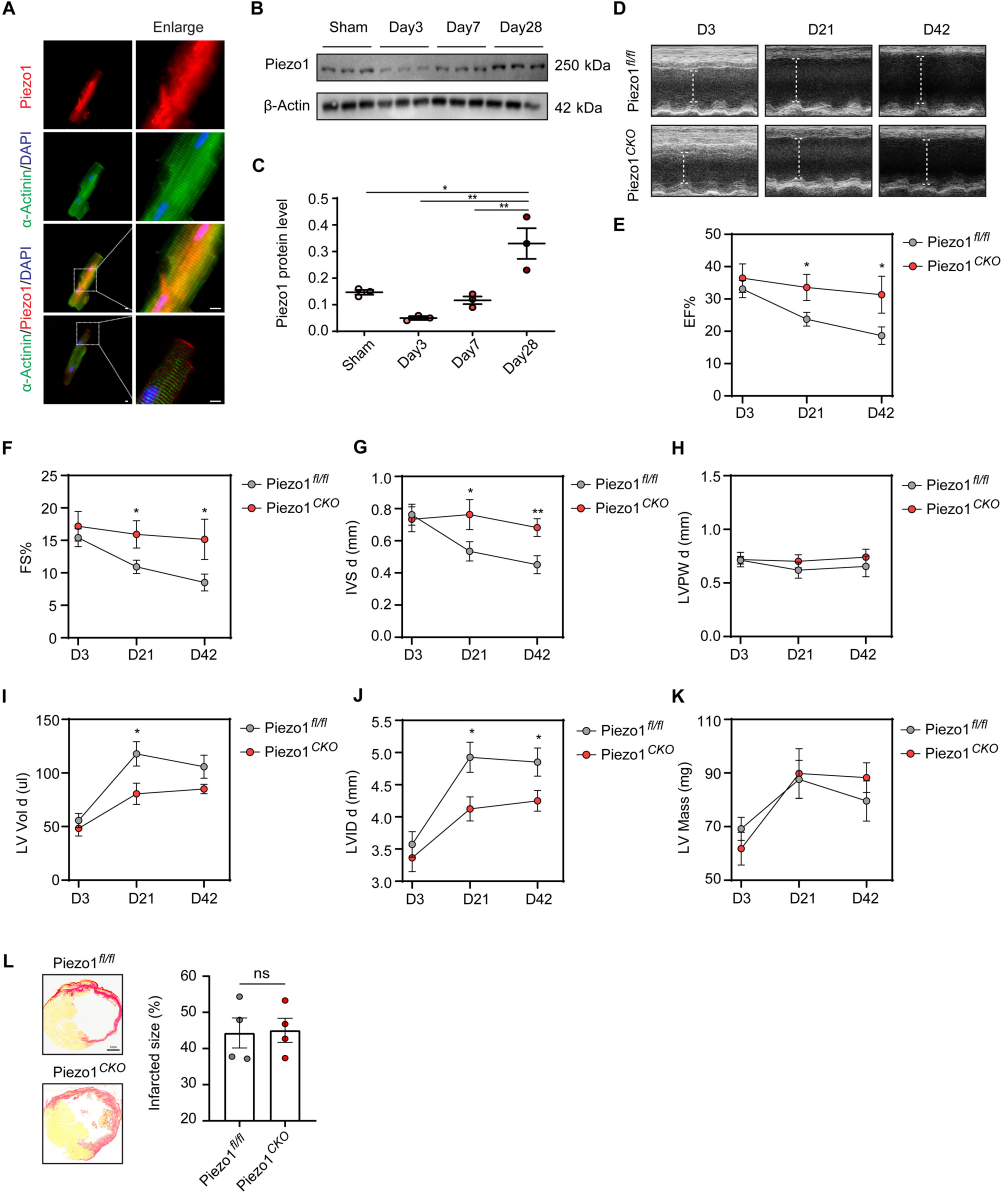
Cardiomyocyte deficiency of Piezo1 improves cardiac function after MI in mice. (A) Representative images of immunofluorescence costaining of Piezo1 (in red) and α-actinin (in green) in the isolated adult mouse cardiomyocyte; scale bar represents 5 μm. (B) Piezo1 expression in the border and infarct zone of myocardium after MI was detected via Western blotting, with β-actin used as a loading control. (C) Piezo1 protein expression was quantified according to the immunoblot. (D) Representative M-mode echocardiographic images of Piezo1*^fl/fl^* and Piezo1*^Cko^* mice at days 3, 21, and 42 after MI, respectively. (E) Left ventricular EF from the echocardiographic data from day 3 to day 42 after MI. (F to H) Left ventricular FS, the interventricular septal thickness at diastole (IVS d), and the thickness of left ventricular posterior wall at diastole (LVPW d) from the echocardiographic data from day 3 to day 42 after MI. (I to K) Left ventricular volume at diastole (LV Vol d), left ventricular internal diameter at diastole (LVID d), and left ventricular mass (LV Mass) from the echocardiographic data from day 3 to day 42 after MI. *n* = 10 for the Piezo1*^fl/fl^* group and *n* = 8 for the Piezo1 Cko group. (L) Representative images of Sirius red staining and quantified infarcted size of Piezo1*^fl/fl^* and Piezo1*^Cko^* mice at day 42 after MI; scale bar represents 1 mm. *n* = 4 for each group. *P < 0.05, **P < 0.01.

Cardiomyocytes are intertwined by intercalated discs and contract in synchronism to meet circulatory demands. Impairment of cell–cell interaction induces cardiomyopathy and pump failure [17]. Interestingly, we found that Piezo1 is mainly located at the intercalated discs and T-tubules by immunofluorescence staining (Fig. 2A), implying a potential role in regulating ventricular function. In the setting of the mouse MI model, Piezo1 expression decreased on day 3 after MI but gradually rose from 7 to 28 days after MI (Fig. 2B and C). To explore the contribution of cardiomyocyte Piezo1 during cardiac remodeling after MI, we crossed Piezo1*^fl/fl^* mice to Myh6-MerCreMer transgenic mice, generating cardiomyocyte conditional Piezo1 knockout mice (Piezo1*^Cko^*). Littermates not carrying the *Myh6-Cre* transgene (Piezo1*^fl/fl^*) served as controls. The deletion efficiency was confirmed in isolated adult cardiomyocytes (Fig. S1B). The basic cardiac function between the 2 groups showed no significant difference (Table S2). Assessed by echocardiography, cardiac Piezo1 deletion showed a protective role on cardiac function as evidenced by the slight decrease of ejection fraction (EF) from 36.43% ± 4.44% to 31.31% ± 5.73% between the 3rd day to the 6th week after MI, while EF prominently declined from 33.04% ± 2.69% to 18.64% ± 2.73% in the Piezo1*^fl/fl^* group (Fig. 2D and E and Table S3). In accompany with the alteration of EF, the fractional shortening (FS) was significantly maintained in the Piezo1*^Cko^* group, while it was obviously declined in the Piezo1*^fl/fl^* group (Fig. 2F). Notably, the interventricular septal thickness at diastole (IVS d) was preserved in the Piezo1*^Cko^* group but reduced in the Piezo1*^fl/fl^* group (Fig. 2G), while the thickness of the left ventricular posterior wall (LVPW d) was comparable between the 2 groups (Fig. 2H). Eventually, within prolonged time, the Piezo1*^fl/fl^* mice exhibited enlarged left ventricular volume at diastole (LV Vol d) and left ventricular internal diameter (LVID d) after MI (Fig. 2I and J). However, the left ventricular mass (LV Mass) was comparable between Piezo1*^Cko^* and Piezo1*^fl/fl^* mice subjected to infarction (Fig. 2K).

Fibrosis distorts the myocardial architecture by depositing excessive extracellular matrix (ECM) and exacerbates cardiac dysfunction following MI. To assess the extent of fibrosis after Piezo1 knockout, Sirius red staining was used. It was notable that the area of fibrotic scarring did not differ between the Piezo1*^fl/fl^* group and the Piezo1*^Cko^* group (Fig. 2L). Meanwhile, the mRNA expression levels of fibrotic genes, including *MMP9*, *ACTA2*, and *TGFBR2*, were comparable between the 2 groups (Fig. S2A to C).

### Piezo1 regulates cardiomyocyte Ca^2+^ handling in mice

**Fig. 3.**
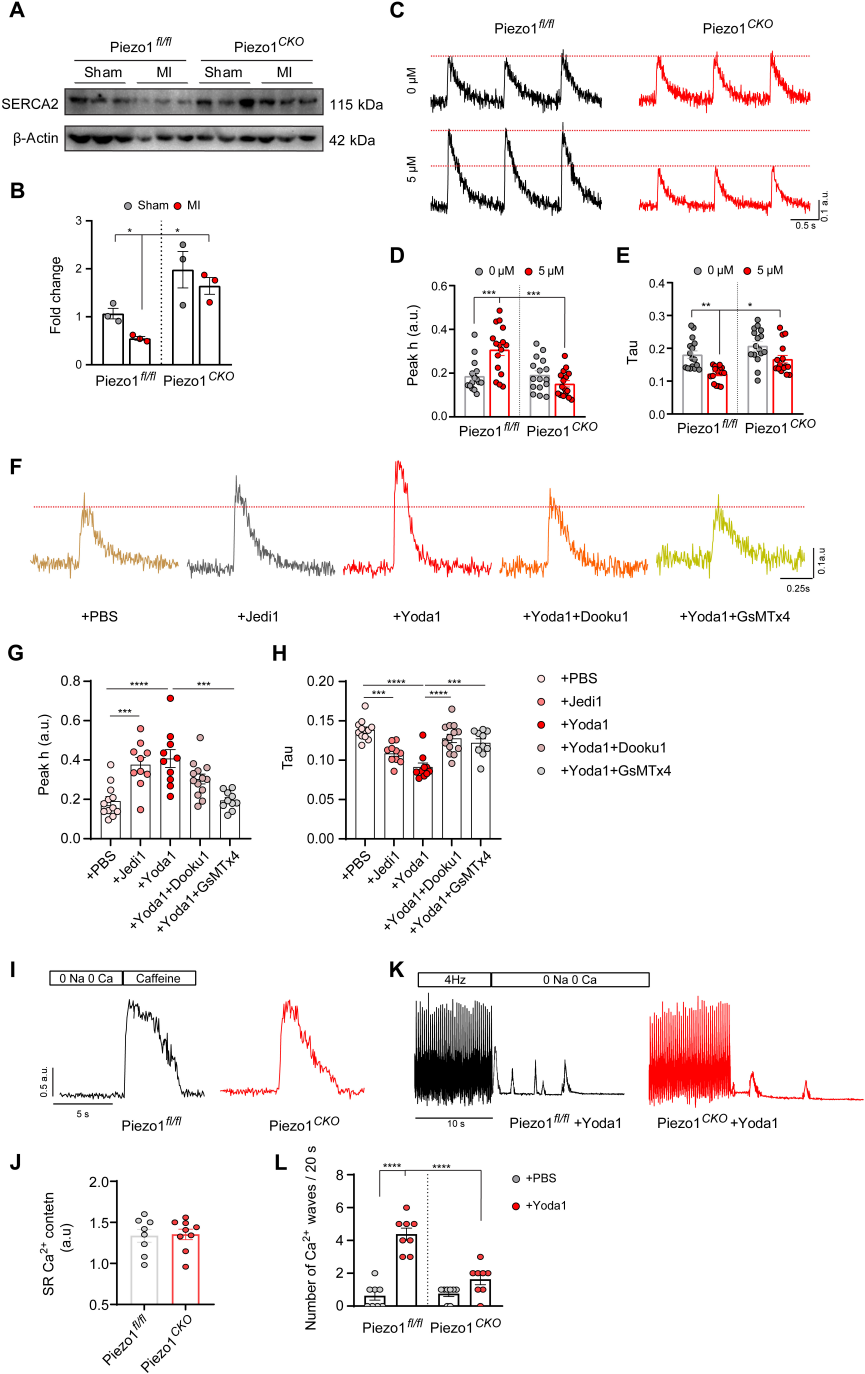
Cardiomyocyte Piezo1 regulates Ca^2+^ transient in mice. (A) SERCA2 expression in the myocardium from Piezo1*^fl/fl^* and Piezo1*^Cko^* mice at day 42 after MI. (B) Quantified result of SERCA2 protein expression according to the immunoblots. (C) Representative Ca^2+^ transient traces from Piezo1*^fl/fl^* and Piezo1*^Cko^* cardiomyocytes in the absence/presence of Yoda1 (5 μM) stimulation; scale bar in horizontal represents 0.5 s and that in vertical represents 0.1 a.u. (arbitrary unit). (D and E) Quantified height from baseline to peak (peak h) and time constant of intracellular Ca^2+^ concentration decline (Tau) according to the Ca^2+^ transient traces (*n* = 16 cells from Piezo1*^fl/fl^* and Piezo1*^Cko^* mice). (F) Representative Ca^2+^ transient traces from adult mouse cardiomyocyte under the stimulation of Jedi1 (500 μM), Yoda1 (5 μM), Yoda1 (5 μM) + Dooku1 (20 μM), and Yoda1 (5 μM) + GsMTx4 (2.5 μM) stimulation; scale bar in horizontal represents 0.25 s and that in vertical represents 0.1 a.u. (G and H) Quantified height from baseline to peak (peak h) and time constant of intracellular Ca^2+^ concentration decline (Tau) according to the Ca^2+^ transient traces (*n* = 12 cells from PBS group; *n* = 10 cells from Jedi1 group, Yoda1 group, and Yoda1 + GsMTx4 group; *n* = 13 cells from Yoda1 + Dooku1 group). (I) Representative traces of calcium signaling from cardiomyocytes paced at 1 Hz and the following caffeine (10 mM) superfusion. (J) Quantified SR Ca^2+^ content (*n* = 8 cells from Piezo1*^fl/fl^* mice and *n* = 9 cells from Piezo1*^Cko^* mice). (K) Representative traces of spontaneous Ca^2+^ release. (L) Number of Ca^2+^ waves in 20 s (*n* = 8 cells from Piezo1*^fl/fl^* mice and Piezo1*^Cko^* mice). *P < 0.05, **P < 0.01, ***P < 0.001, ****P < 0.0001.

Given that cardiac deletion of Piezo1 significantly preserved cardiac function without affecting ECM deposition after MI as shown above, we sought to examine whether Piezo1 deletion reserves myocyte contractility. First, the expression level of sarcoplasmic reticulum (SR) Ca^2+^-adenosine triphosphatase (ATPase) 2 (SERCA2) responsible for myocyte contraction was investigated. In accordance with the preserved cardiac function, the SERCA2 expression was dramatically retained in Piezo1*^Cko^* mice at week 6 after MI (Fig. 3A and B). SERCA2-mediated SR Ca^2+^ uptake is critical in regulating the excitation–contraction coupling of cardiomyocytes [18]. To further explore whether Piezo1 activation affects this process, the Ca^2+^ transient was assessed. At the myocyte level, basal Ca^2+^ transient amplitude and time constant of intracellular Ca^2+^ concentration decline (Tau), indicators of SR Ca^2+^-ATPase function, SR Ca^2+^ content, and Na^+^/Ca^2+^ exchange function, were not significantly altered between Piezo1*^Cko^* and Piezo1*^fl/fl^* groups. However, activation of Piezo1 by its agonist Yoda1 exhibited an obvious elevation of Ca^2+^ transient amplitude and a faster decline of intracellular Ca^2+^ concentration, which was highly suppressed in the Piezo1*^Cko^* mice (Fig. 3C to E). As well as Yoda1, another novel agonist of Piezo1, Jedi1 [19] exhibited a moderate elevation of Ca^2+^ transient amplitude. Furthermore, Yoda1-induced increment of intracellular Ca^2+^ could be blocked by the Yoda1 analog Dooku1 or the Piezo1 inhibitor GsMTx4 (Fig. 3F to H).

To comprehensively understand the role of Piezo1 in regulating calcium handling in cardiomyocytes, we measured the intra-SR Ca^2+^ content following the train of stimulation of calcium transient. After reaching 1 Hz, the SR Ca^2+^ stores were opened with a rapid application of caffeine (10 mM). The total SR Ca^2+^ content was comparable between Piezo1*^Cko^* and Piezo1*^fl/fl^* cardiomyocytes (Fig. 3I and J).

It has been described that the hypersensitive and “leaky” ryanodine receptor (RyR2) could generate spontaneous Ca^2+^ release [20]. To assess this feature, we used a protocol as previously described [21]. Briefly, after a train of stimuli, pacing is stopped, and the bath medium is rapidly changed for a solution without Na^+^ and Ca^2+^ (0 Na–0 Ca) to rule out the influences of sarcolemmal channels (Na^+^ current, Ca^2+^ current) and the sodium/calcium exchanger. Thus, the Ca^2+^ release evoked is the consequence of activated RyR2 channels in diastole. Spontaneous Ca^2+^ release events were observed in both Piezo1*^Cko^* and Piezo1*^fl/fl^* cardiomyocytes. However, the number of waves witnessed was significantly higher in the Piezo1*^fl/fl^* group pretreated with Yoda1 compared with the Piezo1*^Cko^* group (Fig. 3K and L).

### Cardiomyocyte deficiency of Piezo1 ameliorates ventricular electrical instability after MI in mice

**Fig. 4.**
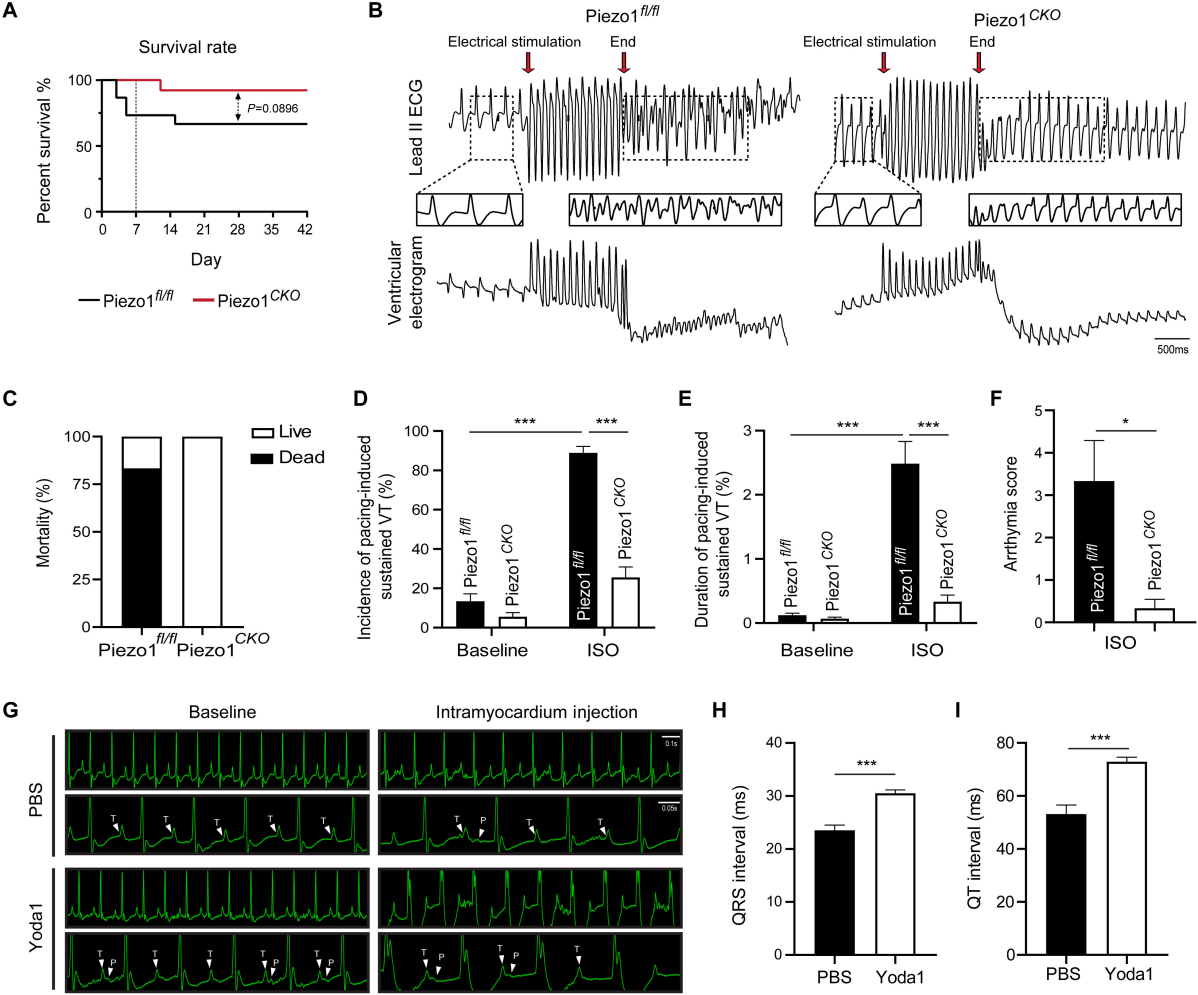
Piezo1 attributes to ventricular electrical instability after MI in mice. (A) Kaplan–Meier curve showing the survival rate between Piezo1*^fl/fl^* (*n* = 15) and Piezo1*^Cko^* (*n* = 13) groups, which were monitored daily until day 42 after MI. (B) Representative lead II and ventricular electrograms of Piezo1*^fl/fl^* and Piezo1*^Cko^* mice undergoing PES at day 42 after MI; red arrow points at the beginning of the programmed electric pulse; scale bar represents 500 ms. (C) Induced mortality of Piezo1*^fl/fl^* and Piezo1*^Cko^* mice after PES after MI. (D and E) Incidence and duration of pacing-induced sustained VT between Piezo1*^fl/fl^* and Piezo1*^Cko^* mice in the absence/presence of isoprenaline (ISO) stimulation after MI. (F) Quantified arrhythmia score of Piezo1*^fl/fl^* and Piezo1*^Cko^* mice in the presence of ISO stimulation after MI; *n* = 6 for each group. (G) Representative lead II electrograms of mice with direct myocardium injection of PBS or Yoda1 (5 μM); white arrows indicated T and p waves; scale bars represent 0.1 and 0.05 s. (H and I) Quantified QRS and QT intervals from PBS and Yoda1 stimulation groups; *n* = 5 for each group. *P < 0.05, ***P < 0.001.

Notably, the survival rate was significantly decreased to 73.33% in the Piezo1*^fl/fl^* mice within 1 week and continuously dropped to 66.67% up to 6 weeks (die/total 5/15), while the Piezo1*^Cko^* mice mostly survived throughout the observation (die/total 1/13) (Fig. 4A). Malignant arrhythmia is a key primary cause of death in mice subjected to MI [22]. To further examine whether Piezo1 was involved in electrical instability after MI, programmed electrical stimulation (PES) was utilized. PES induces sustained ventricular tachycardia (VT), which can be aggravated by isoproterenol (ISO) injection. The survival rate was only 16.67% in Piezo1*^fl/fl^* mice with MI when subjected to PES (Fig. 4B and C). Interestingly, Piezo1*^Cko^* mice with MI showed high resistance to PES (Fig. 4B and C). Furthermore, ISO greatly increased the incidence and duration of sustained VT and arrhythmia score in Piezo1*^fl/fl^* mice with MI (Fig. 4D to F). Piezo1*^Cko^* and Piezo1*^fl/fl^* mice did not exhibit differences in heart rate, electrocardiogram (ECG) parameters, at baseline, or in response to ISO after MI modeling (Table S4). Besides, direct activation of Piezo1 by its specific agonist Yoda1 through intramyocardial injection led to a typical fishhook-like depression in the ST-T segment in mouse ECG (Fig. 4G), suggesting abnormalities in repolarization. The QRS and QT intervals were also obviously prolonged in the group under Yoda1 stimulation (QRS: Yoda1 30.53 ± 0.63 ms vs. PBS 23.52 ± 1.01 ms, QT: Yoda1 72.88 ± 1.77 ms vs. PBS 53.16 ± 3.38 ms) (Fig. 4H and I), suggesting that Piezo1 activation was attributed to the electrical instability of the heart.

### Piezo1 regulates Ca^2+^ signaling and alters action potential in mouse cardiomyocytes

**Fig. 5.**
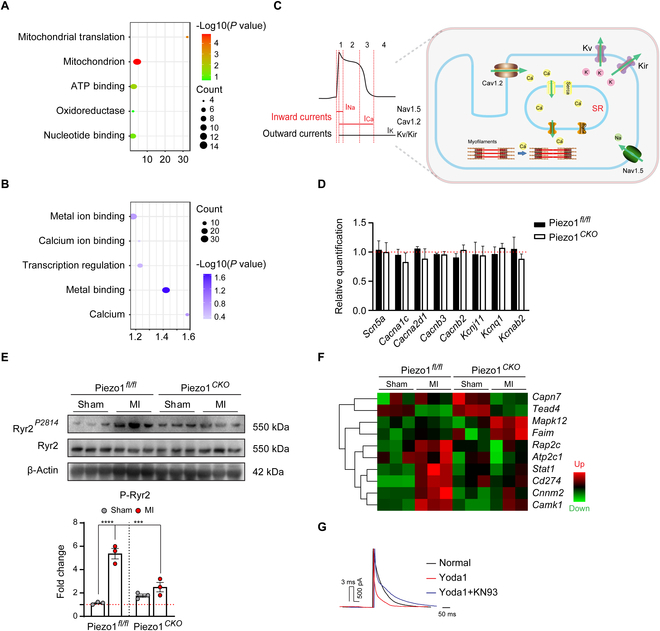
Piezo1 regulates Ca^2+^-modulated signaling and alters action potential in mouse cardiomyocytes. (A and B) Gene Ontology (GO) annotation of the differentially expressed proteins in myocardium from Piezo1*^fl/fl^* and Piezo1*^Cko^* mice at day 42 after MI, which were screened by mass spectrometry. (C) Graph abstract of the primary ion channels involved in action potential formation. (D) Quantified results of the alterations in the primary ion channels in myocardium from Piezo1*^fl/fl^* and Piezo1*^Cko^* mice at day 42 after MI, which were screened by mass spectrometry. (E) Phosphorylated RyR2 expression in myocardium from Piezo1*^fl/fl^* and Piezo1*^Cko^* mice at day 42 after MI (*n* = 3 for each group), and quantified results according to the immunoblots. (F) Heatmap of the alterations in Ca^2+^-related/modulated molecules in myocardium from Piezo1*^fl/fl^* and Piezo1*^Cko^* mice at day 42 after MI, which were screened by mass spectrometry. (G) Representative traces of action potential of isolated adult mouse cardiomyocytes under Yoda1 stimulation (5 μM) and/or CaMKII inhibitor KN93 (2 μM); *n* = 5 cells for each group. *P < 0.05, **P < 0.01, ***P < 0.001, ****P < 0.0001. Capn7, calpain 7; Tead4, tea domain transcription factor 4; *Mapk12*, mitogen-activated protein kinase 12; *Faim*, Fas apoptotic inhibitory molecule; *Rap2c*, Rap2c, member of Ras oncogene family; *Atp2c1*, ATPase secretory pathway Ca^2+^ transporting 1; *Stat1*, signal transducer and activator of transcription 1; *Cd274*, CD274 molecule; *Cnnm2*, cyclin and CBS domain divalent metal cation transport mediator 2; *Camk1*, calcium/calmodulin-dependent protein kinase I.

To profile the proteomes of Piezo1*^fl/fl^* and Piezo1*^Cko^* hearts after MI, mass spectrometry was applied to detect the heart tissue at week 6 after MI. Overall, proteins enriched in mitochondrial translation, adenosine triphosphate (ATP) binding, and oxidoreductase were up-regulated, while proteins enriched in metal ion binding, especially calcium binding, were down-regulated after Piezo1 deletion (Fig. 5A and B). At the myocyte level, currents of sodium, calcium, and potassium channels underlying the ventricular action potential were the molecular mechanisms of arrhythmogenesis (Fig. 5C). However, Piezo1 deletion did not alter the expression of key ion channels in action potential generation and gap junction, including Nav1.5 (SCN5A), Cav1.2 (CACNA1C), Kir6.2 (KCNJ11), Kv7.1(KCNQ1), and connexin43 (GJA1) (Fig. 5D and Fig. S3A to D), but affected the sensitization of the ryanodine receptor (RyR2), which may result in arrhythmias [23]. Phosphorylation of RyR2 at Ser^2814^ [site for calcium/calmodulin-dependent protein kinase II (CaMKII) activation] triggers the increased diastolic SR Ca^2+^ leak and provokes cardiac arrhythmias [24]. In Piezo1*^fl/fl^* mice, the phosphorylated level of RyR2 at Ser^2814^ was enhanced after MI, while it was significantly inhibited in Piezo1*^Cko^* mice (Fig. 5E). Consistently, Ca^2+^-dependent regulators/kinases such as calpain7 (CAPN7) and calcium/calmodulin-dependent protein kinases (CaMKs) were significantly decreased in Piezo1*^Cko^* hearts, while several critical proteins against apoptosis and oxidative stress like Fas apoptotic inhibitory molecule (FAIM) and mitogen-activated protein kinase 12 (MAPK12) were elevated (Fig. 5F), suggesting that Ca^2+^-modulated signaling was suppressed after Piezo1 deletion. By applying the neonatal rat cardiomyocytes, we confirmed that Piezo1 activation by Yoda1 could enhance the activity of CaMKII and calpain in a dose-dependent manner (Fig. S4A and B). Moreover, we found that the action potential of mouse cardiomyocytes was dramatically shortened within Yoda1 stimulation, while it was blocked after KN93 (CaMKII inhibitor) incubation (Fig. 5G).

**Fig. 6.**
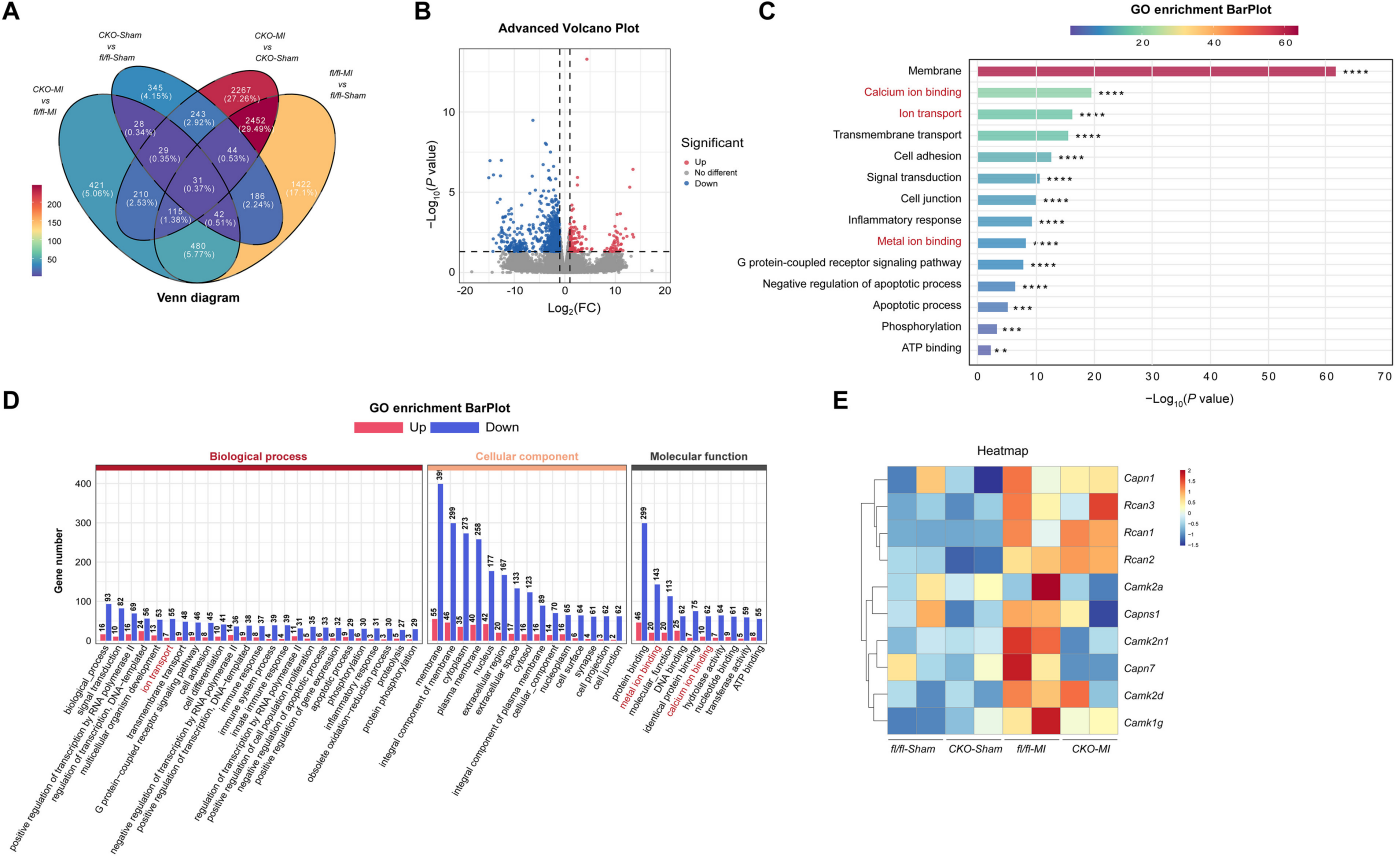
Piezo1 regulates Ca^2+^-modulated signaling at the transcriptional level. (A) Venn diagram of the differentially expressed genes in myocardium from the Piezo1*^fl/fl^* and Piezo1*^Cko^* mice at day 42 after MI or sham groups, which were screened by RNA sequencing. (B) Volcano map of the differentially expressed genes from the Piezo1*^Cko^* and Piezo1*^fl/fl^* hearts after MI operation. (C and D) GO annotation of the differentially expressed genes from the Piezo1*^Cko^* and Piezo1*^fl/fl^* hearts after MI operation. (E) Heatmap of the alterations in Ca^2+^-related/modulated molecules in myocardium from Piezo1*^fl/fl^* and Piezo1*^Cko^* mice at day 42 after MI. *Capn1*, calpain 1; *Rcan3*, regulator of calcineurin 3; *Rcan1*, regulator of calcineurin 1; *Rcan2*, regulator of calcineurin 2; *Camk2a*, calcium/calmodulin-dependent protein kinase II alpha; *Capns1*, calpain small subunit 1; *Camk2n1*, calcium/calmodulin-dependent protein kinase II inhibitor1; *Capn7*, calpain 7; *Camk2d*, calcium/calmodulin-dependent protein kinase II delta; *Camk1g*, calcium/calmodulin-dependent protein kinase IG.

To further confirm whether Piezo1 affects the Ca^2+^-related signaling at the transcriptional level, we applied RNA sequencing to profile the transcriptomes of Piezo1*^fl/fl^* and Piezo1*^Cko^* hearts at week 6 after MI. As shown in the Venn diagram and the volcano map, 1356 genes were differentially expressed between MI-operated Piezo1*^Cko^* and Piezo1*^fl/fl^* groups, including 1095 down-regulated genes and 261 up-regulated genes (Fig. 6A and B). Genes enriched in calcium ion binding, ion transport, and metal ion binding were mostly down-regulated after MI due to Piezo1 deletion (Fig. 6C and D), including CaMKII and calpains (Fig. 6E), which was consistent with the mass spectrometry results.

Taken together, these data suggested that Piezo1 perturbed calcium homeostasis by mediating the intracellular Ca^2+^ overload and increased the activation of Ca^2+^-modulated signaling, CaMKII, and calpain, leading to the enhancement of phosphorylation of RyR2 and further increment of Ca^2+^ leaking, which finally provoked cardiac arrhythmias.

### Piezo1 activation alters properties of action potential in human cardiomyocytes

**Fig. 7.**
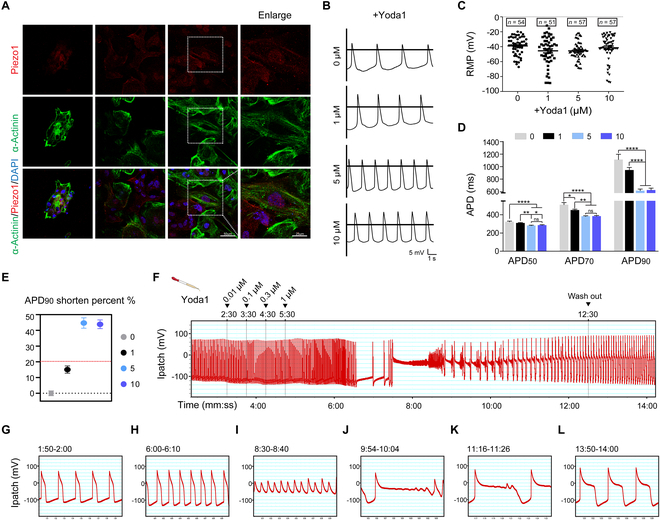
Piezo1 activation triggers cellular arrhythmogenic remodeling in human cardiomyocyte. (A) Representative images of immunofluorescence costaining of Piezo1 (in red) and α-actinin (in green) in cultured hiPSC-CMs at the 25th day; 4′,6-diamidino-2-phenylindole (DAPI)-stained nuclei are shown in blue; scale bars represent 50 and 25 μm (Enlarge). (B) Representative traces of action potential of hiPSC-CMs under a gradient of concentration of Yoda1 stimulation from 0 to 10 μM; the scale bar in horizontal represents 1 s and that in vertical represents 5 mV. (C) Quantified resting membrane potential (RMP) of action potentials of hiPSC-CMs under Yoda1 stimulation from 0 to 10 μM. (D) Quantified action potential duration at 50% (APD_50_), 70% (APD_70_), and 90% (APD_90_) of hiPSC-CMs under Yoda1 stimulation from 0 to 10 μM. (E) Quantified shorted percent of APD_90_ of hiPSC-CMs under Yoda1 stimulation at 1, 5, and 10 μM compared with 0 μM. (F) Representative traces of hiPSC-CMs under a progressively increasing concentration gradient of Yoda1 stimulation from 0.01 to 1 μM. (G to L) Enlarged traces at the specified period from (F). *P < 0.05, **P < 0.01, ***P < 0.001, ****P < 0.0001.

To further verify the arrhythmogenic effect of Piezo1 in human beings, the human induced pluripotent stem cell-derived cardiomyocytes (hiPSC-CMs) were employed. hiPSC-CM was an ideal approach, which can recapitulate inherited arrhythmia syndromes at the cellular level [25,26]. We first identified the location of Piezo1 by costaining with α-actinin in hiPSC-CMs. Given that hiPSC-CMs exhibit an immature phenotype, Piezo1 was predominantly located at the cytoplasmic membrane (Fig. 7A). Although activation of Piezo1 under Yoda1 stimulation in hiPSC-CMs did not change the resting membrane potential, it distinctively accelerated the action potential rate in a Yoda1 dose-dependent manner (Fig. 7B and C). Meanwhile, action potential durations (APDs) including APD_50_, APD_70_, and APD_90_ were progressively shortened in parallel with the increased Yoda1 concentration, implicating that Piezo1 activation could shorten the repolarization process of action potential (Fig. 7D and Table S5). APD was a critical parameter to assess the arrhythmic risk. Excessive shortened or prolonged action potential with over 20% alteration of APD_90_ has been proven proarrhythmic [27]. Compared with hiPSC-CMs without Yoda1 stimulation, the hiPSC-CMs stimulated within 1 μM Yoda1 showed a 15.07% ± 2.32% reduction of APD_90_. The reduction degree kept climbing to 44.64% ± 3.30% and 43.85% ± 2.77% within 5 and 10 μM Yoda1 stimulation, respectively (Fig. 7E). Exaggerated repolarization instability was also observed in hiPSC-CMs with Yoda1 stimulation (5 and 10 μM) (Fig. S5), showing a remarkably aggravation of arrhythmogenic risk.

### Piezo1 activation triggers cellular arrhythmogenic remodeling in human cardiomyocytes

Triggered activity resulting from the premature activation by afterdepolarizations is associated with shortening or prolonging in APDs [28]. Early afterdepolarizations (EADs), developing before full repolarization at phase 2 or phase 3 of cardiac action potential, is one of the major mechanisms driving arrhythmogenesis in diverse etiologies. Surprisingly, persistent activation of Piezo1 by a progressively increasing concentration gradient of Yoda1 resulted in EADs and further continuous triggered activity in hiPSC-CMs (Fig. 7F). APD was rapidly shortened within 1 min after Yoda1 concentration increased to 1 μM (Fig. 7G and H). Then, persistent triggered activity was observed followed by EADs (Fig. 7I). As time went on, the incidence of triggered EADs gradually decreased, which was supposed to be attributable to the exhausted extracellular Ca^2+^ or Piezo1 deactivation (Fig. 7J and K). Action potentials ultimately returned to normal after Yoda1 washout (Fig. 7L). These data suggested that activation of Piezo1 results in triggered activity, which underlies the arrhythmogenesis induced after MI.

## Discussion

Although the mortality has greatly declined after application of early reperfusion in patients with acute MI, the survivors remain at high risk of SCD over the subsequent weeks to years despite extensive pharmacotherapy of secondary prevention. The prevalence of SCD is especially high in the first year following MI, which is a vulnerable period of heart [29]. During the remodeling process after MI, wall thickness is heterogeneously distributed over the ventricle, resulting in a regional modulation of a mechanical stimulus so that regional strain alters. These structural heterogeneities further attribute to stretch-induced ventricular arrhythmia [30]. However, the underlying mechanism is poorly understood. In the present study, we uncovered a previously unrecognized role of the mechanosensitive ion channel Piezo1 in the electrical remodeling after MI and its arrhythmogenic effect on cardiomyocyte through Ca^2+^ handling, showing dramatic prospects in therapeutic approaching SCD and heart failure within cardiac injury.

Piezo1 is a stretch-activated cation channel with preference to Ca^2+^. It is a critical sensor regulating mechano-chemo-transduction in vasculature, while its role in heart, especially under pathological condition, is unclear. We observed that Piezo1 majorly locates at the intercalated disk and T-tubules. Deletion of Piezo1 in cardiomyocyte preserved the long-term cardiac function and survival rate after MI. Notably, this effect seemed less associated with fibrotic remodeling evidenced by the comparable fibrotic gene expression and scarring area. Cardiac contractility is determined by the cyclic movement of calcium among the extracellular space, cytoplasm, and SR. This Ca^2+^ handling attributes for the excitation–contraction coupling in cardiomyocytes. SERCA2 is a critical protein involved in sequestration of Ca^2+^ into SR during diastole, linking contractile force with excitation–contraction coupling. The reduced SERCA2 gene expression and protein level are commonly witnessed in ischemic or failing myocardium, attributing to dysfunction of SR Ca^2+^ uptake and weakened cardiac contraction [31]. We observed that transient activation of Piezo1 in cardiomyocyte accelerates SR Ca^2+^ uptake on account for the increased intracellular Ca^2+^ flux. However, Piezo1 deletion in cardiomyocyte preserved the long-term SERCA2 expression after MI, suggesting that sustained activation of Piezo1 after MI down-regulates SERCA2 expression. The inhibitory effect may blame for Ca^2+^ overload-induced imbalanced homeostasis of ROS signaling [12], which has been proven to reduce SERCA2 expression [32]. In addition, SERCA2 was capable of interacting directly with Piezo1 by strategically binding to its linker region and further suppressing Piezo1-mediated mechanosensitive currents [33]. On the reverse side, it was suspected that deletion of Piezo1 may preserve the activity of SERCA2 through structural variation.

In addition to SR Ca^2+^ uptake dysfunction, Ca^2+^ overload triggers spontaneous Ca^2+^ leak from SR as well. Given that RyR2 in SR has a finite open probability even at diastolic [Ca^2+^]i, Ca^2+^ will leak out of SR, resulting in the occurrence of intracellular Ca^2+^ waves so that Ca^2+^ spreads beyond the original sites and elicits arrhythmogenic afterdepolarizations [18]. Our data illustrated that Piezo1 activation after MI promotes the phosphorylation of RyR2, which is evidenced to vary the sensation of RyR2 and increase diastolic SR Ca^2+^ leakage [34]. Potential mechanism could be attributed from Piezo1-enhanced activity of CaMKII, which is crucial to phosphorylate RyR2 and contribute to a further destabilization of RyR2 [35,36]. At the cellular level, Piezo1 activation triggered arrhythmogenic remodeling through remarkably shortening APD and inducing EADs, which occurs late in phase 3 of action potential. An abbreviated APD permits normal Ca^2+^ release from SR. However, when the [Ca^2+^]_i_ keeps rising until the membrane potential is negative to the equilibrium potential for the Na^+^/Ca^2+^ exchanger (NCX), *I*_NCX_ will be activated, causing membrane depolarization. These late EADs are clinically relevant with tachycardia including atrial tachycardia, VT, and ventricular fibrillation [37]. In compliance with our data, triggered activity defined by continuous EADs could be seen under the sustained but not evanescent activation of Piezo1. Furthermore, as a nonselective cationic channel, Piezo1 conducts Na^+^ influx as well. Raised intracellular Na^+^ could consequently activate the reverse mode of NCX to increase [Ca^2+^]_i_ [38]. In summary, the above inferences pointed the possible mechanism in the occurrence of arrhythmia linked to Piezo1*^Cko^* mice after MI.

Our study demonstrated that Piezo1 is critically involved in arrhythmogenic remodeling after MI through Ca^2+^ regulation and revealed it as a new potential anti-arrhythmic target in the treatment of MI and heart failure.

## Methods

The detailed methods are available in the Supplementary Materials.

### Human heart tissue

Three nondiseased human hearts were obtained from 3 healthy donors who died from brain-stem bleeding. Four failing hearts were obtained from patients with end-stage DCM who underwent cardiac transplantation. The hearts were collected and flushed with ice-cold cardioplegic solution. Then, parts of the left ventricular myocardium were frozen in liquid nitrogen for protein extraction, and the other parts were fixed in formaldehyde solution for immunohistochemical staining. All subjects were duly informed, and written consent was provided by patients or their relatives. All the human studies were approved by the Human Research Ethics Committee of the Second Affiliated Hospital, Zhejiang University School of Medicine and were performed under the principles of the Declaration of Helsinki.

### Animal model of MI

Piezo1*^fl/fl^* mice (*Piezo1*^tm2.1Apat^/J) and Myh6-Cre transgenic mice (A1cf^Tg(Myh6-cre/Esr1*)1Jmk^/J) were purchased from Jackson Laboratory. Cardiac-specific Piezo1 knockout mice (Piezo1*^Cko^*) were created by crossing Piezo1*^fl/fl^* mice with Myh6-Cre mice. Littermates not carrying the *Myh6-Cre* transgene (Piezo1*^fl/fl^*) served as controls. Inducible deletion of Piezo1 in cardiomyocyte was performed in 4-week-old mice by intraperitoneal administration of tamoxifen (75 mg/kg/day, consistent for 5 days).

Eight-week-old mice were subjected to MI modeling as previously described [39]. Briefly, mice were anesthetized with sodium pentobarbital (50 mg/kg intraperitoneally) and then ventilated via tracheal intubations connected to a rodent ventilator. Acute MI was performed by permanent ligation of the left anterior descending coronary artery (LAD) using a 7-0 nylon suture. Sham surgery was performed exactly as above but without LAD ligation. For tissue harvesting, mice were euthanized with sodium pentobarbital (80 mg/kg intraperitoneally) following cervical dislocation, and then hearts were quickly excised after thoracotomy.

All animals were fed a standard laboratory diet and maintained with a 12:12-h light/dark cycle. All animal studies were performed in compliance with guidelines of the Institutional Animal Care and Use Committee at Zhejiang University School of Medicine (zju201308-1-01-085) and the National Institutes of Health (NIH) *Guide for the Care and Use of Laboratory Animals* (NIH Publication No. 85-23, revised 1996).

### Statistical analysis

Data were presented as means ± SEM. For data with small samples, the Mann–Whitney *U* test was used between the 2 groups. Survival data were exhibited by Kaplan–Meier curves and analyzed by the log-rank test. The other data were confirmed normal distribution by the Kolmogorov–Smirnov test. Statistical differences were then determined by Student’s *t* test for comparison between 2 groups and analysis of variance (ANOVA) followed by Bonferroni’s multiple comparison test for comparison among 3 or more groups. *P* < 0.05 was considered statistically significant. Statistical calculations were carried out using GraphPad Prism 8.0.

## Data Availability

All data could be acquired from M.X. upon requests.
