# Supplementary material for: Cardiac Piezo1 Exacerbates Lethal Ventricular Arrhythmogenesis by Linking Mechanical Stress with Ca2+ Handling After Myocardial Infarction
Source: Research (Wash D C). 2023 Jun 9;6:0165. doi: 10.34133/research.0165 (PMC10255393; doi:10.34133/research.0165)
Supplement: Supplementary 1 — Figs. S1 to S5 [file research.0165.f1.docx]

**Supplemental Material**

**Methods**

**Echocardiography**

Mouse transthoracic two-dimensional echocardiography was performed using Vevo 2100 system (VisualSonics, Canada). The echocardiography was repeated at 3 days, 3 weeks, 6 weeks and 8 weeks post-MI, respectively. Mice were anesthetized by inhalation of isoflurane (1-1.5%). M-mode interrogation was performed in the parasternal long-axis view at the level of the papillary muscles. LV end-diastolic dimension, LV end-systolic dimension, and septal and LV posterior wall thicknesses were determined and used to calculate the percentage of ejection fraction (EF) and fractional shortening (FS).

**Programmed Electrical Stimulation**

Programmed electrical stimulation (PES) was performed in Piezo1*^Cko^* and Piezo1*^fl/fl^* mice at the 6^th^ week post MI. As previously described^1^, mice were anesthetized with sodium pentobarbital (50mg/kg intraperitoneally) and their limbs were taped onto ECG electrodes. Then the right jugular vein was exposed by separating subcutaneous tissues. A 1.1F octapolar catheter with 4 pairs of electrical leads (iWorx, USA) was inserted through the vein into the right ventricle. The catheter was connected with the Intracardiac Electrical Potential Recording and Measurement System (iWorx, USA). The ventricular pacing train was set at a fixed basic cycle length (BCL, 90ms). The S1-S2 interval was progressively reduced by 2-ms in each pacing train from 70ms to 40ms. Between each stimulation protocol, there was a recovery period of ~30 seconds. The incidence of reproducible sustained ventricular tachycardia (SVT, episode>10 consecutive beats of VT) was determined with or without isoproterenol (0.5mg/kg intraperitoneally). The arrhythmia score was calculated according to the standard previously described^2^: 0=no ventricular arrhythmia; 1=isolated ventricular extrasystoles (VES); 2=VES in bigeminy; 3=couplets; 4=VT, VT was characterized as ≥3 consecutive VES.

**Isolation of Adult Mouse Cardiomyocyte and Neonatal Rat Cardiomyocyte**

A simplified, Langendorff-free method for isolation of viable cardiomyocyte from adult mouse was performed^3^. Briefly, mice were sacrificed and the heart with clamped aortic arch was completely separated from the thoracic cavity. The following perfusion of EDTA buffer in the right ventricle, and the digestion buffer in the left ventricle were performed in turn. Not until the digestion was apparent, the heart tissue was then pulled gently into ~1mm^3^ pieces and stop buffer was added. Cell suspension was filtered through 100μm strainer and myocytes gravity settled for 20 min. The pellets were resuspended in calcium re-introduction buffer and prepared for further experiments.

Neonatal rat cardiomyocytes were isolated following the instruction of the neonatal heart dissociation kit (130-098-373, Miltenyi BioTec). Cells were cultured in DMEM/F12 (Gibco) with 10% FBS and 0.1mM BrdU to inhibit fibroblast proliferation.

**Differentiation and Culturing of Human Induced Pluripotent Stem Cell-Derived Cardiomyocytes (hiPSC-CMs)**

Human iPS cells (BCMi001-A iPSCs) were obtained from Dr. Jean J. Kim (Baylor College of Medicine Human Stem Cell Core, USA). The hiPSCs were grown in TeSR^TM^-E8^TM^ medium (Stemcell Technologies, Canada). Differentiation of hiPSCs was undertaken by using the STEMdiff™ Cardiomyocyte Differentiation Kit (Stemcell Technologies). On the 8^th^ day of differentiation, small areas of beating cardiomyocytes were visible, at which point the medium was changed into STEMdiff™ Cardiomyocyte Maintenance Medium (Stemcell Technologies) for the remainder of the study. At day 25, the cells were passaged for patch clamp experiments.

**Patch clamp experiments**

Patch-clamp experiments were performed following a previously described method^4^. For action potential (AP) recording, the pipette solution contained 2.7M KCl, and the Tyrode solution served as bath solution, which consisted of NaCl (140mM), KCl (5.4mM), CaCl_2_ (1.8mM), MgCl_2_ (1mM), HEPES (10mM) and glucose (10mM), PH 7.4. The pipette resistance was ~60-90 MΩ. APs were recorded using the current-zero with the gap-free model in pClamp10.0 software (Molecular Devices, Canada). The indexes including resting membrane potential, APD_50_, APD_70_ and APD_90_ (the time intervals required to reach 50%, 70% and 90% of repolarization), dV/dt_max_, AP triangularity, and beat-to-beat instability were measured using LabChart8.0 software (AD Instruments, New Zealand).

**Measurement of Calcium Transient and SR Ca^2+^ Content**

Measurement of intracellular calcium was performed in freshly isolated left ventricular cardiomyocytes using the Myocyte Calcium and Contractility System (IonOptix Corporation, USA). Briefly, adult cardiomyocytes were labeled with Fura-2-AM (1μmol/L, Thermo Scientific, USA) for 20 min and precipitated on an open-perfusion chamber mounted on the stage of an upright microscope (Leica, German) for 10 min. Then the attached cells were perfused with Tyrode’s solution. Cadiomyocytes were stimulated at 5V, 1Hz using a Myopacer Cell Stimulator (IonOptix Corporation, USA). Dual excitation (at 340/380nm, F1 and F0) was delivered using OptoLED light sources and emission light was collected at 510nm. Simultaneous tracing of calcium transients were recorded and analyzed using IonOptix software. The measured parameters included calcium amplitude and calcium transient decay (Tau).

The SR Ca^2+^ content was evaluated at the end of the train of stimulation describes above. After reaching 1 Hz, the total SR Ca^2+^ content was detected by a rapid application of caffeine (10mM). The data were analyzed using IonOptix software.

**Detection of Spontaneous Ca^2+^ Release**

Cardiomyocytes were labeled with Fura-2-AM (1μmol/L) for 20 min and then stimulated at 5V, 4Hz using the Myopacer Cell Stimulator. After a train of stimuli, pacing was stopped and the medium was quickly changed into a Na^+^ and Ca^2+^-free Tyrode solution to rule out the influences of sarcolemmal channels. Spontaneous Ca^2+^ release tracs were recorded using IonOptix software.

**Mass Spectrometry and Bioinformatic Analysis**

Cryopreserved myocardiums were prepared for mass spectrometry by Westlake Omics (Hangzhou) Biotechnology Co., Ltd. (China). Briefly, peptides were extracted from the myocardium tissue and labeled with TMTpro 16plex label reagents. LC-MS/MS with the LC system coupled to an Orbitrap Exploris 480 mass spectrometer (Thermo Fisher, San Jose, USA), which equipped with a FAIMS Pro™ (Thermo Fisher Scientific™, San Jose, USA) , in data dependent acquisition (DDA) mode. For each acquisition, peptides were loaded onto a precolumn (3 μm, 100 Å, 20 mm*75 μm i.d.) at a flowrate of 6 μL/min for 4 min and then injected using a 45 min LC gradient (from 3% to 28% buffer B) at a flowrate of 300 nL/min (analytical column, 1.9 μm, 120 Å, 150 mm*75 μm i.d.). Buffer A was 2% ACN, 98% H2O containing 0.1% FA, and buffer B was 98% ACN in water containing 0.1% FA. All reagents were MS grade. MS/MS experiments were performed with a resolution at 30,000, normalized AGC target of 200%, and max IT of 100 ms. The turbo-TMT and advanced Peak Determination were enabled, isolation window was set to 0.7 Da and first mass was set to 110 m/z.

Results were fit in the database using Proteome Discoverer (Ver2.4, Thermo Fisher). Gene Ontology (GO) enrichment analysis of differentially expressed proteins was implemented by the clusterProfiler R package.

**RNA Sequencing and Analysis**

Total RNA was isolated from mouse myocardium using Trizol reagen. The total RNA quantity and purity were analysis of Bioanalyzer 2100 and RNA 6000 Nano LabChip Kit. After total RNA was extracted, mRNA was purified from total RNA (5ug) using Dynabeads Oligo (dT) with two rounds of purification. Following purification, the mRNA was fragmented into short fragments using divalent cations under elevated temperature. Then the cleaved RNA fragments were reverse-transcribed to create the cDNA by SuperScript™ II Reverse Transcriptase. The average insert size for the final cDNA libraries were 300±50 bp. At last, we performed the 2×150bp paired-end sequencing (PE150) on an Illumina Novaseq™ 6000 (LC-Bio Technology CO., Ltd., Hangzhou, China). The sequence quality was verified using FastQC (http://www.bioinformatics.babraham.ac.uk/projects/fastqc/, 0.11.9). including the Q20, Q30 and GC-content of the clean data. After that, a total of G bp of cleaned, paired-end reads were produced. The raw sequence data have been submitted to the NCBI Gene Expression Omnibus (GEO) datasets with accession number GSE230042.

**Sirius Red Staining**

Mouse heart samples were harvested and embedded in paraffin, cut into 4-μm thick sections from mid-papillary to apex (500-μm intervals) and stained with Sirius red (Solaribio, China) for 1 hour followed by 95% ethyl alcohol washing. The images were obtained with a microscope (Leica, German). The percentage of infarct area was calculated using Imaging Pro software.

**Immunohistochemistry**

Human heart samples were embedded in Paraffin and sliced into 4-μm sections. Immunohistochemical staining was performed using the antibody for Piezo1 (Cat No. NBP1-78537, Novus). Quantification of Piezo1 immunopositive area was determined by

Image Pro software indicated by positive staining area/total area.

**Immunofluorescence Staining**

Isolated adult mouse cardiomyocytes or cultured hiPSC-CMs were fixed in 4% paraformaldehyde. The cells were co-stained with primary antibodies for Piezo1 (Cat No. NBP1-78537, Novus) and α-actinin (Cat No. ab9465, Abcam). Cy3-conjugated goat anti-rabbit and FITC-conjugated goat anti-mouse antibodies (Abcam) were applied as secondary antibodies. Nuclei were stained by DAPI. Images were obtained using a fluorescence microscope (Leica, German).

**Western Blot**

Proteins were extracted from mouse heart tissues using RIPA solution (Beyotime, China) within a protease inhibitor cocktail (Roche). 20μg of each protein sample was separated via SDS-PAGE and electrotransferred onto PVDF (polyvinylidene fluoride) membranes. Following blockade with PBST (phosphate-buffered solution with Tween-20) containing 5% BSA, membranes were incubated with primary antibodies against the following molecules overnight at 4°C: Piezo1 (Cat No. NBP1-78537, Novus), Serca1/2 (Cat No: A010-21AP, Badrilla), Ryr2 (Cat No: ab2827, Abcam) and Ryr2^P2814^ (Cat No: A010-31AP, Badrilla). Antigen and antibody complexes were detected with an ECL protocol using HRP conjugated IgG as secondary antibodies. Immunoblots were quantified using Image Laboratory 2.0 software.

**Detection of CaMKII and Calpain activity**

Neonatal rat cardiomyocytes were incubated within 1h-incubation of Yoda1 with a gradient concentration from 1μM to 10μM. Cells were then collected for activity detection according to the instructions, respectively. CaMKII activity was measured using CaMKII-β/γ/δ (Phospho-Thr287) Cell-Based ELISA Kit (LifeSpan BioSciences, LS-F1677). Calpain activity was analyzed using the Calpain Activity Assay Kit (Abcam, ab65308). Cells were handled following the instructions and the final fluorescence units were measured (Ex/Em = 400/505 nm) to acquire the original data of Calpain activity. Meanwhile, the optical density was measured in OD 450nm to acquire the original data of CaMKII activity. Data were then normalized to the sample protein concentration and shown as the relative Calpain and CaMKII activity.

**Real-Time PCR**

Total RNA was extracted from mouse heart tissues using Trizol reagent (Invitrogen). 1000ng of total RNA was reversely transcribed into cDNA by using PrimeScript RT reagent Kit (Takara, Japan). The resulting cDNA was then subjected to real-time PCR using SYBR Premix Ex Taq II kits (Takara, Japan) and performed by Lightcycle480 II (Roche). β-actin was used as an internal loading control. mRNA levels of target genes were normalized to β-actin levels using the relative threshold cycle method. All primer information was listed in **Supplemental Table I**.

**Supplemental Table I**

| **Primers for Mouse (5’ to 3’)** | |
| --- | --- |
| β-actin Forward | TGTGGATCAGCAAGCAGGAG |
| β-actin Reverse | TGCGCAAGTTAGGTTTTGTC |
| MMP9 Forward | TCCCCAAAGACCTGAAAACC |
| MMP9 Reverse | CTGCTTCTCTCCCATCATCTG |
| ACTA2 Forward | CTTCGTGACTACTGCCGAGC |
| ACTA2 Reverse | AGGTGGTTTCGTGGATGCC |
| TGFBR2 Forward | ATGTGGAAATGGAAGCCCAGA |
| TGFBR2 Reverse | TGCAGGACTTCTGGTTGTCG |
| CACNA1C Forward | CCTGCTGGTGGTTAGCGTG |
| CACNA1C Reverse | TCTGCCTCCGTCTGTTTAGAA |
| KCND2 Forward | CACTCTCAAGGGCTGCGTAT |
| KCND2 Reverse | TCGTTTGTCTGCTCGTTGGT |
| SCN5A Forward | GGAGTACGCCGACAAGATGT |
| SCN5A Reverse | ATCTCGGCAAAGCCTAAGGT |
| GJA1 Forward | CCAAGGAGTTCCACCACTTTG |
| GJA1 Reverse | CCATGTCTGGGCACCTCTCT |

**Supplemental Table II**

| **Baseline** | **EF%** | **FS/%** | **LV Vol d/μl** | **LV Mass/mg** |
| --- | --- | --- | --- | --- |
| **Piezo1*^fl/fl^*** | 84.05±3.77 | 52.73±4.21 | 20.04±1.93 | 77.22±7.38 |
| **Piezo1*^Cko^*** | 84.98±2.30 | 52.46±2.85 | 16.97±2.32 | 86.48±9.91 |

**Supplemental Table III**

|  | EF% | FS/% | IVS d/mm | LVPW d/mm | LV Vol d/ul | LVID d/mm | LV Mass/mg |
| --- | --- | --- | --- | --- | --- | --- | --- |
| Day3 | | | | | | | |
| Piezo1*^fl/fl^* | 33.04±2.69 | 15.40±1.38 | 0.76±0.07 | 0.71±0.04 | 55.75±6.67 | 3.57±0.20 | 69.15±4.31 |
| Piezo1*^Cko^* | 36.43±4.44 | 17.18±2.28 | 0.73±0.08 | 0.72±0.07 | 48.29±7.18 | 3.36±0.22 | 61.76±6.10 |
| Day21 | | | | | | | |
| Piezo1*^fl/fl^* | 23.72±2.14 | 10.93±1.02 | 0.53±0.06 | 0.62±0.08 | 117.79±11.36 | 4.93±0.23 | 87.64±7.09 |
| Piezo1*^Cko^* | 33.57±4.07 | 15.93±2.10 | 0.76±0.09 | 0.70±0.06 | 80.53±9.90 | 4.13±0.19 | 89.80±9.26 |
| Day42 | | | | | | | |
| Piezo1*^fl/fl^* | 18.64±2.73 | 8.50±1.30 | 0.45±0.06 | 0.65±0.10 | 105.85±10.81 | 4.85±0.22 | 79.59±7.51 |
| Piezo1*^Cko^* | 31.31±5.73 | 15.17±3.12 | 0.68±0.06 | 0.74±0.07 | 85.10±4.40 | 4.25±0.16 | 88.29±5.54 |

**Supplemental Table IV**

| **Parameters** | **Baseline** | |  | **ISO** | |
| --- | --- | --- | --- | --- | --- |
|  | **Piezo1*^fl/fl^*** | **Piezo1*^Cko^*** |  | **Piezo1*^fl/fl^*** | **Piezo1*^Cko^*** |
| **Heart Rate/min** | 483.17±10.11 | 476.50±15.95 |  | 541.67±14.98 | 538.83±19.47 |
| **QT interval/ms** | 81.50±2.12 | 82.17±2.28 |  | 77.50±1.15 | 76.17±1.28 |
| **PR interval/ms** | 39.83±1.28 | 38.17±1.07 |  | 36.67±1.52 | 37.33±1.02 |

**Supplemental Table V**

| **Yoda1(μM)** | **0** | **1** | **5** | **10** |
| --- | --- | --- | --- | --- |
| **N**  **Beating Rate/min** | 54  15.42±0.39 | 51  23.54±0.26 | 57  34.09±0.58 | 57  33.75±0.82 |
| **APD_50_/ms** | 322.27±8.11 | 309.60±4.57 | 281.18±4.38 | 286.37±4.90 |
| **APD_70_/ms** | 509.98±23.24 | 451.56±9.53 | 382.59±6.15 | 385.20±6.59 |
| **APD_90_/ms** | 1112.79±84.44 | 945.14±42.08 | 616.05±25.08 | 624.88±31.76 |
| **Triangulation**  **(APD_90_-APD_30_)/ms** | 925.39±80.24 | 750.76±46.10 | 441.51±25.94 | 438.10±33.45 |

**Supplemental Figures**


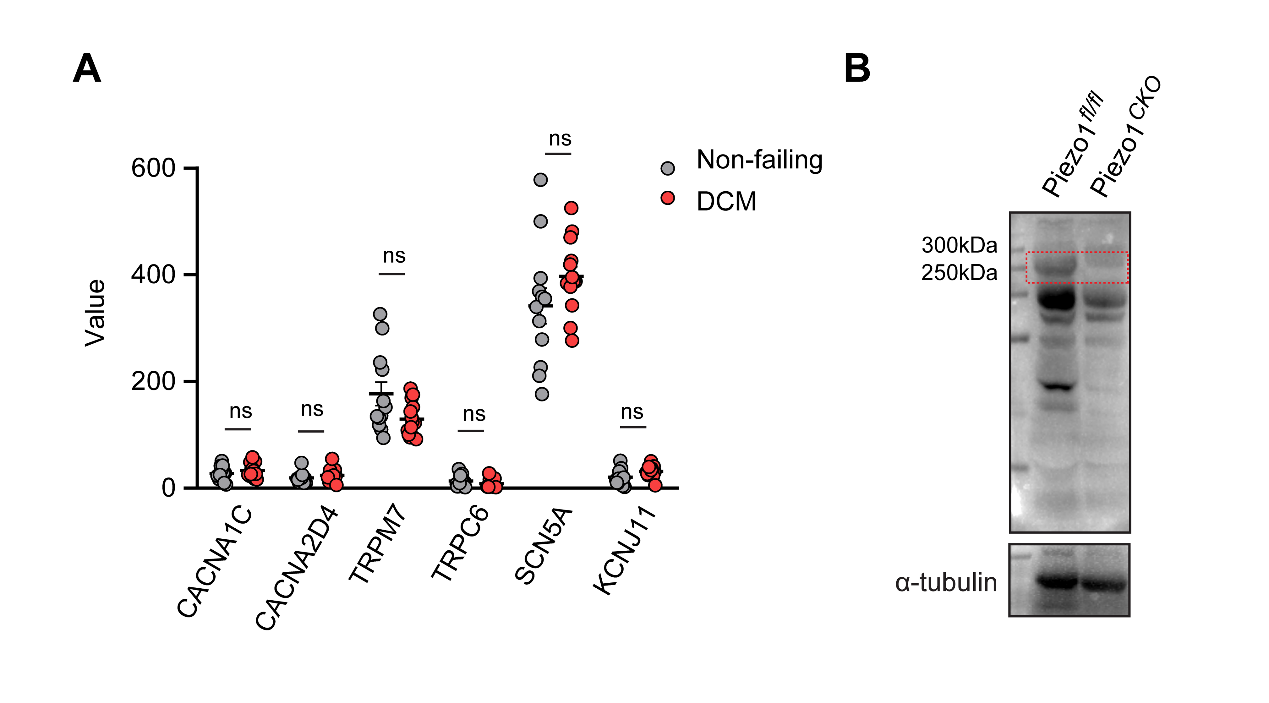


**Supplemental Figure 1. A)** Quantified value of typical ion channel RNA expressions in non-failing human hearts and hearts from patients with dilated cardiomyopathy (DCM) according to the RNA-sequencing data from GSE29819. **B)** Piezo1 knockout efficiency in isolated adult mouse cardiomyocyte was detected by western blotting, with α-tubulin used as a loading control.


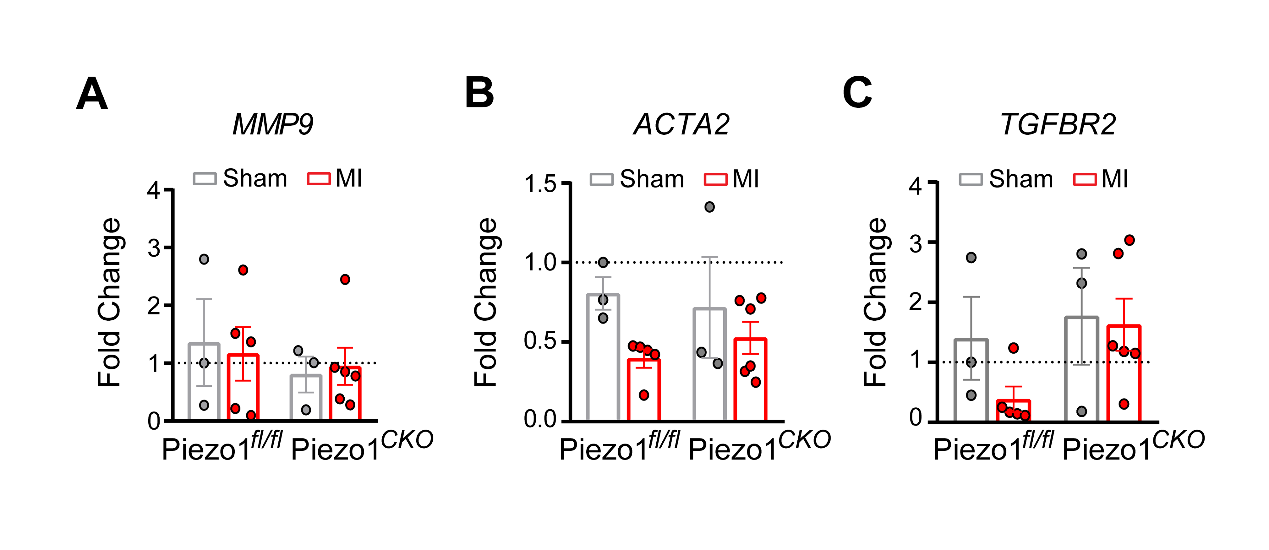


**Supplemental Figure 2. A-C)** Relative expression of *Mmp9*, *Acta2* and *Tgfbr2* in the myocardium from Piezo1*^fl/fl^* and Piezo1*^Cko^* mice at day 42 post-MI were analyzed by real-time PCR.


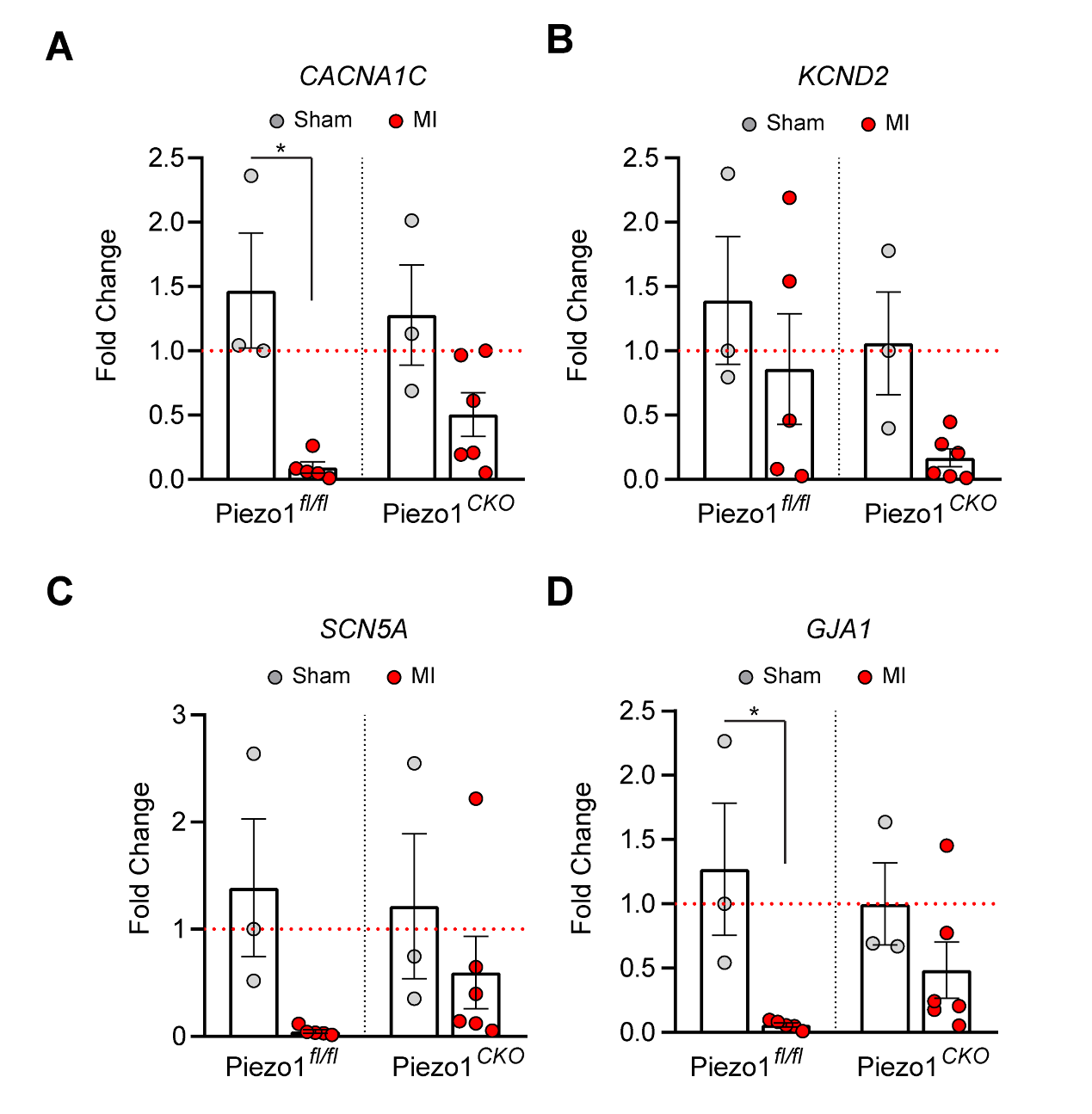


**Supplemental Figure 3. A-D)** Relative expression of *CACNA1C*, *KCND2*, *SCN5A* and *GJA1* in the myocardium from Piezo1*^fl/fl^* and Piezo1*^Cko^* mice at day 42 post-MI were analyzed by real-time PCR.

**Supplemental Figure 4. A)** Detection of CaMKII and Calpain activities in neonatal rat cardiomyocytes following 1h-incubation of Yoda1 within a concentration of 0. 1, 5, 10μM, respectively. *P<0.05, **<0.01.
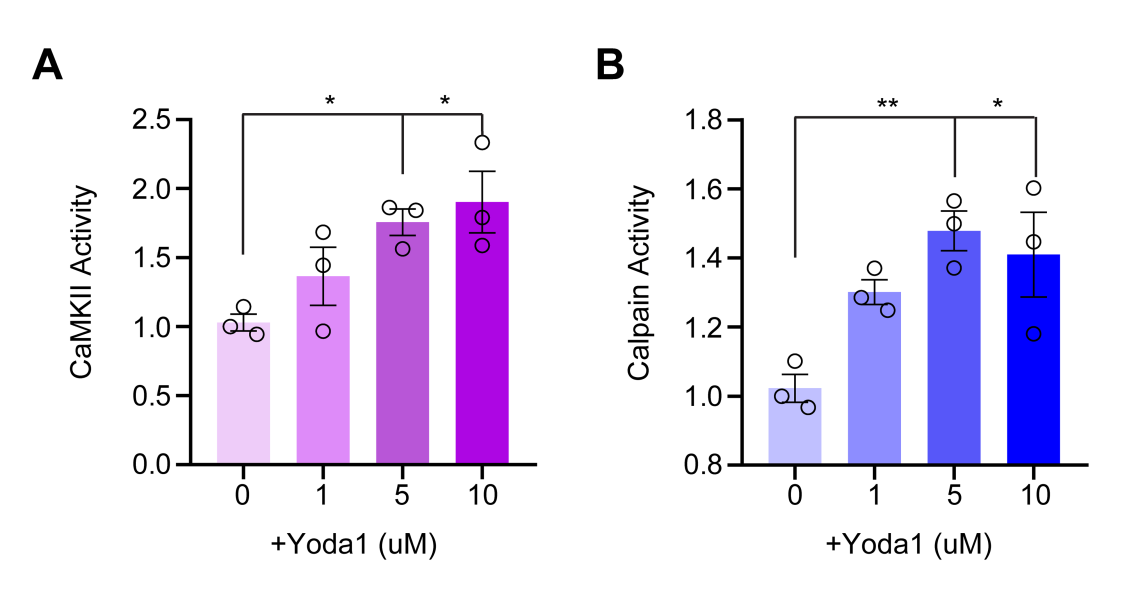


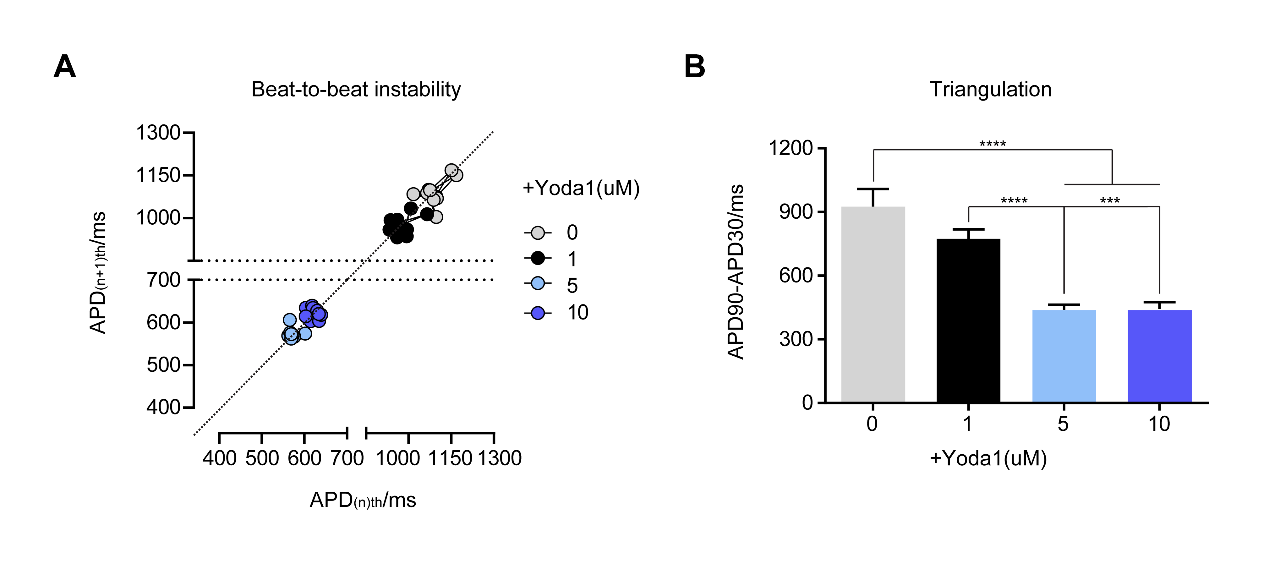


**Supplemental Figure 5. A)** Beat-to-beat instability of action potential of human iPSC-CMs under Yoda1 stimulation were shown by Poincare plots. **B)** Triangulation of action potential of human iPSC-CMs under Yoda1 stimulation was calculated by APD_90_ minus APD_30_. ***P<0.001, ****<0.0001.

**References:**

**1.** Li N, Wehrens XH. Programmed electrical stimulation in mice. *J Vis Exp.* 2010(39).

**2.** Sadredini M, Manotheepan R, Lehnart SE, Anderson ME, Sjaastad I, Stokke MK. The oxidation-resistant CaMKII-MM281/282VV mutation does not prevent arrhythmias in CPVT1. *Physiol Rep.* 2021;9(18):e15030.

**3.** Ackers-Johnson M, Li PY, Holmes AP, O'Brien SM, Pavlovic D, Foo RS. A Simplified, Langendorff-Free Method for Concomitant Isolation of Viable Cardiac Myocytes and Nonmyocytes From the Adult Mouse Heart. *CIRC RES.* 2016;119(8):909-920.

**4.** Zhang J, Wilson GF, Soerens AG, Koonce CH, Yu J, Palecek SP, Thomson JA, Kamp TJ. Functional cardiomyocytes derived from human induced pluripotent stem cells. *CIRC RES.* 2009;104(4):e30-e41.
